# The link between immunity and life history traits in scleractinian corals

**DOI:** 10.7717/peerj.628

**Published:** 2014-10-30

**Authors:** Jorge H. Pinzón C., Lindsey Dornberger, Joshuah Beach-Letendre, Ernesto Weil, Laura D. Mydlarz

**Affiliations:** 1 Department of Biology, University of Texas Arlington, Arlington, TX, USA; 2 Department of Marine Sciences, University of Puerto Rico, Mayagüez, PR, USA

**Keywords:** Coral disease, Constitutive and innate immunity, Hermaphrodite, Gonochoric, Colony morphology, Trade-off, Resource allocation, Scleractinia, Caribbean corals, Biological traits

## Abstract

Immunity is an important biological trait that influences the survival of individuals and the fitness of a species. Immune defenses are costly and likely compete for energy with other life-history traits, such as reproduction and growth, affecting the overall fitness of a species. Competition among these traits in scleractinian corals could influence the dynamics and structural integrity of coral reef communities. Due to variability in biological traits within populations and across species, it is likely that coral colonies within population/species adjust their immune system to the available resources. In corals, the innate immune system is composed of various pathways. The immune system components can be assessed in the absence (constitutive levels) and/or presence of stressors/pathogens (immune response). Comparisons of the constitutive levels of three immune pathways (melanin synthesis, antioxidant and antimicrobial) of closely related species of Scleractinian corals allowed to determine the link between immunity and reproduction and colony growth. First, we explored differences in constitutive immunity among closely related coral species of the genus *Meandrina* with different reproductive patterns (gonochoric vs. hermaphrodite). We then compared fast-growing branching vs. slow-growing massive *Porites* to test co-variation between constitutive immunity and growth rates and morphology in corals. Results indicate that there seems to be a relationship between constitutive immunity and sexual pattern with gonochoric species showing significantly higher levels of immunity than hermaphrodites. Therefore, gonochoric species maybe better suited to resist infections and overcome stressors. Constitutive immunity varied in relation with growth rates and colony morphology, but each species showed contrasting trends within the studied immune pathways. Fast-growing branching species appear to invest more in relatively low cost pathways of the immune system than slow-growing massive species. In corals, energetic investments in life-history traits such as reproduction and growth rate (higher energy investment) seem to have a significant impact on their capacity to respond to stressors, including infectious diseases and coral bleaching. These differences in energy investment are critical in the light of the recent environmental challenges linked to global climate change affecting these organisms. Understanding physiological trade-offs, especially those involving the immune system, will improve our understanding as to how corals could/will respond and survive in future adverse environmental conditions associated with climate change.

## Introduction

The maintenance of an effective immune system is costly to organisms, but it is necessary to prevent and/or fight infections (Lochmiller & Deerenberg, 2000; Rolff & Siva-Jothy, 2003). Interactions between physiological traits and their resulting trade-offs are highly influential in the strategies organisms use to maximize resources (Leuzinger, Willis & Anthony, 2012). Limited resource availability forces trait competition, affecting the physiological functions, such as reproduction, growth and immunity (Williams, 1966; Stearns, 1989; Hall & Hughes, 1996), potentially affecting species fitness (Sandland & Minchella, 2003; Martin, Weil & Nelson, 2008; Graham et al., 2010; van der Most et al., 2010).

Impacts on immunity due to differential resource allocation are common in nature. Temperate birds, for example, increase their body mass during winter affecting their immune functions (Moreno-Rueda, 2011), a trade-off also observed during mating season. Environmental factors impact the distribution of energy across traits (Nogueira, Baird & Soares, 2004; Finstad et al., 2010; Catalan et al., 2012), forcing immunity to be plastic to environmental changes (Ardia, Parmentier & Vogel, 2010). Trade-offs, not involving immunity, have been observed in vertebrates (Nebel et al., 2011; Horrocks et al., 2012) as well as terrestrial (Fuller et al., 2011; Triggs & Knell, 2011; Catalan et al., 2012) and marine invertebrates (Matranga et al., 2000; Samain, 2011; Coates et al., 2012). In scleractinian corals, the interplay between immunity and other biological traits remains elusive.

Scleractinian corals are key in the development, construction and sustainability of coral reefs (Bryant et al., 1998; Spalding, Raviliuous & Green, 2001). The success of corals as reef builders is mostly due to multiple reproductive strategies (Harrison & Wallace, 1990; Harrison, 2011) and variable modular growth (Chappell, 1980; Filatov et al., 2010). Sexual reproduction in corals is complex, with species showing different reproductive patterns (hermaphroditism and gonochorism), different developmental modes (broadcasting and brooding) and different gametogenic cycles and spawning timings (Harrison, 2011). Colony growth and morphology vary from slow-growing in crustose and massive colonies to fast-growing in branching or columnar colonies (Dullo, 2005; Díaz & Madin, 2010). Reproductive strategy, as well as growth rates and form, are costly and might be important in defining which species of corals (McField, 1999; Knowlton, 2001; Baird & Marshall, 2002; Weil, Croquer & Urreiztieta, 2009a; Weil, Croquer & Urreiztieta, 2009b), and/or individuals within a species (Van Woesik et al., 2012), have higher levels of resistance to environmental stressors and/or pathogenic infections. The overall efficiency of the immune system could then be influenced by the way corals allocate resources to different functions.

Several coral immunity pathways, signaling and effector cascades have been characterized including the melanin synthesis cascade (i.e., prophenoloxidase, phenoloxidase and melanin deposits in cells), antioxidants (i.e., superoxide dismutase, peroxidase and catalase) (Mydlarz et al., 2009; Mydlarz, McGinty & Harvell, 2010; Palmer, Bythell & Willis, 2010; Palmer et al., 2011) and antimicrobial compounds and proteins (Mydlarz et al., 2009; Mydlarz, McGinty & Harvell, 2010; Palmer, Bythell & Willis, 2010; Palmer et al., 2011; Vidal-Dupiol et al., 2011a; Vidal-Dupiol et al., 2011b). The activity of these immune related proteins can be assessed in the absence (constitutive levels) and/or presence of pathogens (immune responses). In corals, constitutive levels and immune responses vary across species and exposure to stressors suggesting each species or population may allocate resources to immunity differently (Palmer, Bythell & Willis, 2010; Palmer et al., 2011).

Determining the effect of biological traits (i.e., energy/resource trade-offs) on immunity is critical to understand how species will thrive under adverse environments and more frequent and diverse pathogenic infections. Recent studies of ecological strategies in corals suggest immune traits, along with other biological factors, can define coral species susceptibility and/or competitive edge (Côté & Darling, 2010; Darling et al., 2012; Putnam et al., 2012). In the present study, we take these analyses one-step further and test two hypotheses about the relationship between immunity and other biological traits. Our first hypothesis is that species with different sexual reproductive patterns (gonochoric vs. hermaphrodite) have similar levels of constitutive immunity. Each reproductive pattern, gonochorism and hermaphroditism, has its own advantages and disadvantages (Hamilton & Axelrod, 1990; Bush, Newman & Koslow, 2004) influencing how resources are allocated to this or other biological traits (i.e., immunity or growth). The Caribbean genus *Meandrina* is ideal to test this hypothesis because these species show contrasting reproductive patterns; *M. jacksoni* is gonochoric while *M. meandrites* and *M. danae* are hermaphrodites (Pinzón & Weil, 2011). Our second hypothesis is that constitutive immunity levels are similar in fast-growing branching corals compared to slow-growing massive species. Constitutive immunity levels were compared between the massive/crustose *Porites astreoides* and its fast growing branching sister species *P. porites*, to test the second hypothesis. While our focus is on Caribbean corals, the trends observed in this study could be extrapolated to similar corals in other locations although testing them is recommended.

## Materials and Methods

### Sample collection

Samples were collected from three different reefs in La Parguera, on the southwest coast of Puerto Rico. A mid-shelf, exposed, fringing reef (Media Luna - 17°56.091N and 67°02.577W), a near shore, semi-protected fringing reef (Enrique - 17°57.336N and 67°02.569W) and an exposed lagoonal patch reef (Laurel - 17°56.501N and 67°03.296W). Small fragments (5 cm^2^) of tissue with skeletal material from healthy looking (no signs of bleaching or disease) massive/crustose species were carefully collected using a hammer and chisel. Samples from the healthy-looking branching species were collected by carefully breaking a small branch end. All samples were stored in individually labeled whirl-pack bags (Fisher Scientific, Waltham, MA), placed in a cooler with ice on the boat, transported to the laboratory, flash frozen in liquid nitrogen, and stored at −80 °C. Samples were shipped in dry ice to the University of Texas in Arlington, where they were stored at −80 °C until further analyses. Samples were collected under the specification of research collection permits to the Department of Marine Science UPRM.

The relationship between sexual pattern and immunity was examined in the *Meandrina* genus. All three Caribbean *Meandrina* spp. broadcast their gametes with separate but overlapping reproductive seasons, starting gametogenesis in mid-summer (June/July northern hemisphere). *M. meandrites* and *M. danae* are hermaphrodites and *M. jacksoni* is gonochoric (Pinzón & Weil, 2011). A total of 56 tissue samples were collected from a similar number of colonies (21 *M. danae*, 20 *M. meandrites*, 15 *M. jacksoni*) in August 2011 when colonies are typically investing energy into gametogenesis, a process that for these species finishes between September and December (Pinzón & Weil, 2011).

The relationship between colony morphology and immunity was tested with *P. astreoides* and *P. porites*. In La Parguera, these species are found in the same habitat; both show hermaphroditic and mixed (i.e., hermaphrodite and gonochoric in the same colony) sexual patterns, brood their larvae, and have similar gametogenetic cycles that overlap for several months, including June and July (Szmant-Froelich, 1986; Chornesky & Peters, 1987; Tomascik & Sander, 1987; Soong, 1991). Clear differences between these species are colony morphology and growth rates; with *P. astreoides* forming massive-encrusting-plate colonies that grow at an average of 4.91 ± 3.4 mm yr^−1^ (mean ± SE), while *P. porites* is branching and grows significantly faster (21.16 ± 13.25 mm yr^−1^) (Soong, 1991; Dullo, 2005). In March 2011, when these species are presumably not investing in sexual reproduction, tissue fragments and branches were collected from 17 *P. astreoides* and 13 *P. porites* apparently healthy colonies.

### Protein extraction and biochemical assays

Protein extractions and enzymatic assays followed protocols previously used to study coral immunity (Mydlarz et al., 2009; Mydlarz, McGinty & Harvell, 2010; Palmer, Bythell & Willis, 2010; Mydlarz & Palmer, 2011; Palmer et al., 2011). Coral tissue was removed from the skeleton using a Paasche artists airbrush with low amounts (5–6 ml) of Tris buffer (100 mM Tris, pH 7.8 + 0.5 mM Dithiothreitol). Tissue slurries were stored in 15 ml plastic falcon tubes at −80 °C, after fast freezing in liquid nitrogen. To extract proteins, the samples were freeze-thawed once, and the slurry homogenized using a tissue homogenizer (Powergen 125; Fisher Scientific, Waltham, MA) for 1 min on ice. The slurry (1 ml) was added to pre-weighted 1.5 ml microfuge tubes for melanin concentration estimates. Protein concentrations were estimated using the RED660 protein assay (G Biosciences, Saint Louis, MO) and standardized to a standard curve of bovine serum albumin. All immune assays were conducted on a Synergy 2 Microplate Reader (Biotek Instruments, Winooski, VT).

The melanin synthesis pathway was examined using two assays: melanin synthesis (putatively prophenoloxidase activity) and melanin concentration. The melanin synthesis assay was performed on 20 µl of the extract, mixed with 20 µl of sodium phosphate buffer (50 mM, pH = 7.0) and 25 µl of trypsin (0.1 mg ml^−1^). The reaction was initiated by adding 30 µl of dopamine (10 mM) as a substrate. Change in absorbance was measured every 30 s at 490 nm for a period of 15 min and activity was calculated during the linear range of the curve (1–5 min) and standardized to mg of protein and presented as change in absorbance mg protein^−1^ min^−1^. Melanin concentration was assessed on the melanin-reserved portion of initial tissue slurry after freeze-drying (VirTis BTK; SP Scientific, Warminster, PA) for 24 h. The resulting dried tissue was weighed and the melanin extracted with 400 µl NaOH (10 M) and vortexed for 20 s. The melanin was allowed to extract at room temperature for 48 h at which time the tissue slurry was centrifuged (90 G) for 10 min. 40 µl of the supernatant were used to determine the absorbance at 410 nm on half volume well plates (Corning-Costar, Tewskbury, MA). Resulting values were standardized to a dose–response curve of commercial melanin (Sigma-Aldrich, Saint Louis, MO).

Oxidative stress was assessed on three enzymes: catalase, superoxide dismutase and peroxidase. Catalase was measured as the change in hydrogen peroxide concentration after mixing 5 µl of the protein extract with 45 µl of sodium phosphate buffer (50 mM, pH 7.0) and 75 µl of 25 mM H_2_O_2_. Samples were loaded on UV transparent plates (Grainer Bio-one, Monroe, NC) and read at 240 nm every 30 s for 15 min. Catalase activity is presented as change in hydrogen peroxide concentration per mg of protein during the first 5 min of the reaction. Superoxide dismutase activity was determined with a commercially available kit (SOD Determination kit #19160; Sigma-Aldrich, Saint Louis, MO) following manufacturer’s instructions. Briefly 10 µl of the protein extract were mixed with 200 µl of the WST working solution from the superoxide dismutase determination kit. To this mixture 10 µl of water and 20 µl of enzyme solution were added and incubated for 20 min at 37 °C. Absorbance at 450 nm revealed activity as compared to standards and untreated samples. Activities were normalized to mg of protein. Peroxidase activity was assessed on 10 µl of extract diluted with 40 µl sodium phosphate buffer (0.01 mM, pH = 6.0) and 25 µl guaiacol (25 mM). Activity was monitored for 15 min, recording the absorbance at 470 nm every 30 s. Peroxidase data was standardized to mg of protein and is presented as change in absorbance mg protein^−1^ min^−1^.

Antimicrobial activity tests on coral protein extracts assessed against *Vibrio alginolyticus* (GenBank # X744690), a strain isolated from colonies showing signs of Caribbean Yellow-Band Disease. Bacteria were grown in salt amended (2.5% NaCl) Luria broth (EMD Chemicals, Gibbson, NJ) for 24 h prior to use in the assay. The resulting bacteria culture was diluted to a final optical density of 0.2 at 600 nm. 140 µl of the culture suspension was added to each well along with 60 µl of the coral extract. To detect possible effects of the media and the airbrushing buffer, controls with 60 µl of Tris buffer (100 mM Tris, pH 7.8 + 0.5 mM DTT) were included on each experimental plate. Plates were incubated in the spectrophotometer for 6 h at 29 °C, determining the absorbance (600 nm) every 10 min. Two points from the logarithmic phase were selected for data analysis for each sample. The change in absorbance during the logarithmic phase was used to determine the doubling time (*Dt*) using the equation:

\begin{eqnarray*} D t=\frac{t i m e}{\left(\frac{(\log (F i n a l A b s)-\log (I n i t i a l A b s))}{\log (2)}\right)} \end{eqnarray*}

The percentage of inhibition was calculated based on the change in absorbance from treated wells from that of the controls.

### Statistical analysis

All statistical analyses were performed in JMP 10.0 (SAS Institute, Cary, NC). All data sets were initially tested to confirm normality and homoscedasticity (Shapiro-Wilks and Levene’s test, respectively). Data sets with a distribution significantly different from normal or non-homoscedastic were transformed using the Box-Cox *Y* transformation. To test our first hypothesis, we used a one-way multivariate analysis of variance (MANOVA) to compare constitutive immunity levels between species with different reproductive strategies. Further differences between average immune activities were tested using one-way analysis of variance (ANOVA) and Tukey-Kramer post hoc test between *Meandrina* spp. (gonochoric vs. hermaphrodites). To test our second hypothesis, a one-way multivariate ANOVA (MANOVA) was used to compare constitutive immunity levels between morphologies (massive vs. branching) within the genus *Porites*, followed by *t*-tests for each of the measured traits. All *p*-values were corrected using the false discovery rate (FDR) method as implemented in JMP and tested with an alpha level of 0.05.

## Results and Discussion

Investment in immunological defenses to reduce risks associated with infections can challenge resources allocated to other important physiological functions such as reproduction and growth (Lochmiller & Deerenberg, 2000; Sandland & Minchella, 2003; Simmons, 2011). Results of this study show that investment in immunity may be affected by investments in reproductive strategies and growth/morphology in Caribbean corals. The interaction between these traits could have an impact on coral’s survival and might be important in maintaining the integrity and persistence of coral reef ecosystems in the face of new disease outbreaks, bleaching, climate change and local environmental stressors. Comparing closely related species that differ in a given trait, like reproduction pattern and colony morphology in corals, is a good first approach towards understanding trends in coral immunity. Even though our findings are restricted to a few Caribbean corals, similar patterns might occur in other genera and/or geographic locations, where sister species have different reproductive patterns and/or morphologies.

### Immunity and reproductive strategy

**Figure 1:**
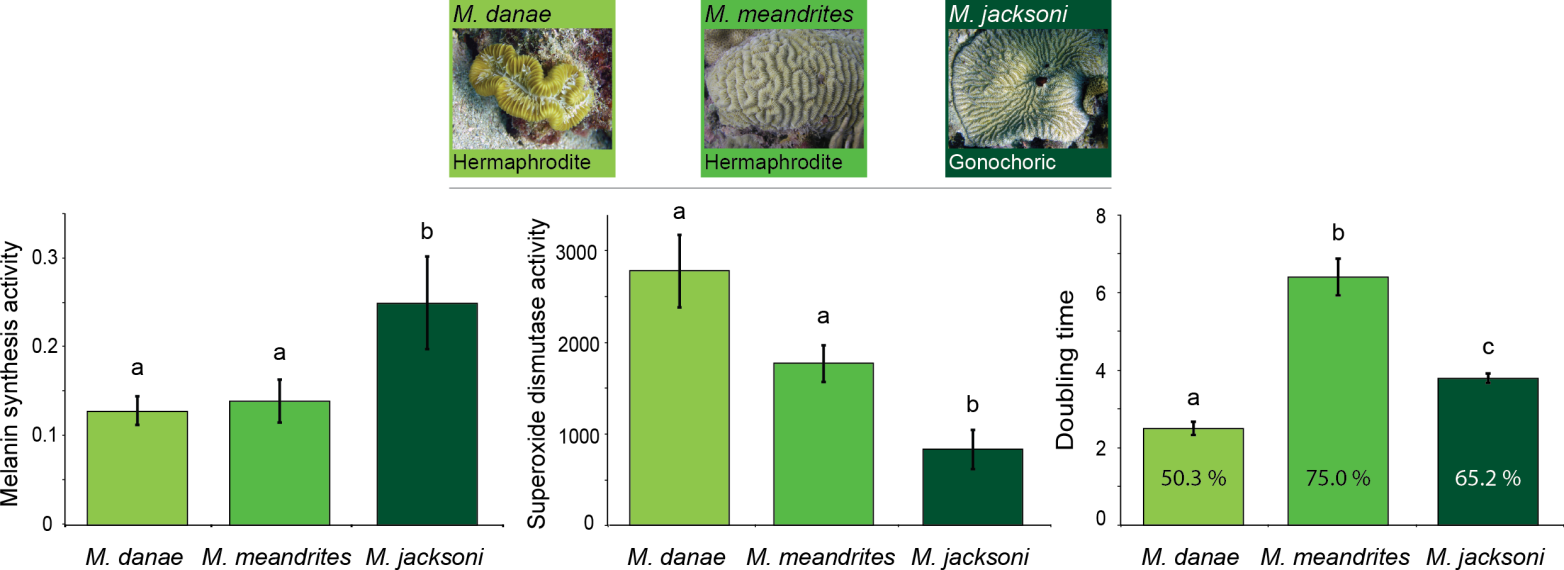
Relation between immunity and reproduction in corals. Mean constitutive immunity among *Meandrina* species with different sexual patterns as determined by melanin synthesis, superoxide dismutase and antibacterial (doubling time and percent inhibition) activity. Letters on the bars indicate significant differences (Tukey post-hoc tests at *p* < 0.05). Data presented as mean ± standard error, for melanin synthesis as Δ absorbance 490 nm mg protein^−1^ min^−1^, for superoxide dismutase as absorbance 450 nm mg protein^−1^ min^−1^, and for doubling time as hours with the percentage of inhibition inside each bar. Antimicrobial data compares growth of *Vibrio alginolyticus* when exposed to coral extract with untreated controls.

Comparisons across immune traits revealed significant differences in constitutive immunity between *Meandrina* species with different reproductive patterns (MANOVA Wilk’s Lambda *F*_12_ = 8.14, *p* < 0.0001; Table 1). Melanin synthesis (putatively prophenoloxidase activity) was significantly lower in the hermaphroditic species (*M. meandrites* and *M. danae*) compared to the gonochoric species (*M. jacksoni*; Fig. 1). In contrast, both hermaphroditic species showed significantly higher superoxide dismutase activities compared to the gonochoric *M. jacksoni* (Fig. 1; Table 1). Antimicrobial activity was also different, with *M. danae* showing the lowest level of inhibition, *M. jacksoni* intermediate values and *M. meandrites* higher inhibitions (Fig. 1; Table 1).

**Table 1:** Constitutive immune levels in phylogenetically close Caribbean corals with different reproductive patterns and colony morphologies. Comparisons of the levels of six constitutive immunity measures between coral species with different reproduction patterns (*Meandrina meandrites* and *M. danae* vs. *M. jacksoni*) and between species with different growth rates and colony morphology (*Porites astreoides* vs. *P. porites*).

	*REPRODUCTION*			*MORPHOLOGY*		
	*Meandrina* spp.			*Porites* spp.		
	*F*	df	*p*	*F*	df	*p*
MANOVA						
Wilk’s lambda	**8.14**	**12**	**0.0001**	**18.00**	**6**	**0.0001**
Univariate tests						
Melanin synthesis	**5.51**	**2**	**0.0101**	**10.27**	**1**	**0.0037**
Melanin concentration	0.70	2	0.5996	**58.58**	**1**	**0.0003**
Superoxide dismutase	**21.41**	**2**	**0.0003**	**43.75**	**1**	**0.0003**
Catalase	**5.87**	**2**	**0.0100**	**11.84**	**1**	**0.0024**
Peroxidase	0.45	2	0.6390	0.11	1	0.7451
Antibacterial	**10.47**	**2**	**0.0003**	**16.50**	**1**	**0.0006**

**Notes.**

Bolded values indicating significant differences.

*F* *F* statistic for the ANOVA and MANOVA analyses df degrees of freedom *p* *p*-values (corrected using False Discovery Rate correction)

Evolutionary and ecological species success is in part driven by their sexual pattern (Alvarez et al., 2005). Since hermaphrodites have to generate both, ova and sperm and prevent self-fertilization, they are likely to allocate more resources to reproduction than to other biological traits (Michiels & Koene, 2006), such as immunity. Our data suggests that corals with different sexual reproductive patterns have different levels of constitutive immunity. As shown in our results, gonochoric corals show higher melanin synthesis activity and lower levels of antioxidants than their conspecific hermaphrodites. Similarly, in plants the immune related enzyme polyphenol oxidase (Mayer, 2006), was more active in female flowers of *Carica papaya* than in hermaphrodite flowers of the same species (Cano et al., 1996). Differences in immune responses across organisms can also be reflected in disease prevalence. Fungal infections in plants showed a lower load of pathogens in dioecious species than in hermaphroditic ones (Williams, Antonovics & Rolff, 2011).

Our findings of differences in immune levels between gonochoric and hermaphrodite Caribbean coral species may have implications to the survivorship of many species as infectious disease outbreaks increase. In the Caribbean, gonochoric species (i.e., *Meandrina jacksoni*, *Montastraea cavernosa*, *Siderastrea siderea*) tend to have fewer diseases than hermaphroditic species (Bruckner & Hill, 2009; Weil & Rogers, 2011). Furthermore, gonochoric species seem to be recruiting more successfully, becoming more common, and defining the new assemblages in declining Caribbean reefs (Bruckner & Hill, 2009; Irizarry-Soto & Weil, 2009; Yakob & Mumby, 2011), which might be underscored by a higher investment in constitutive immunity. Additional factors outside the realm of reproduction may also contribute to the resistance levels of gonochoric species.

### Colony morphology and growth rate effects on constitutive immunity

**Figure 2:**
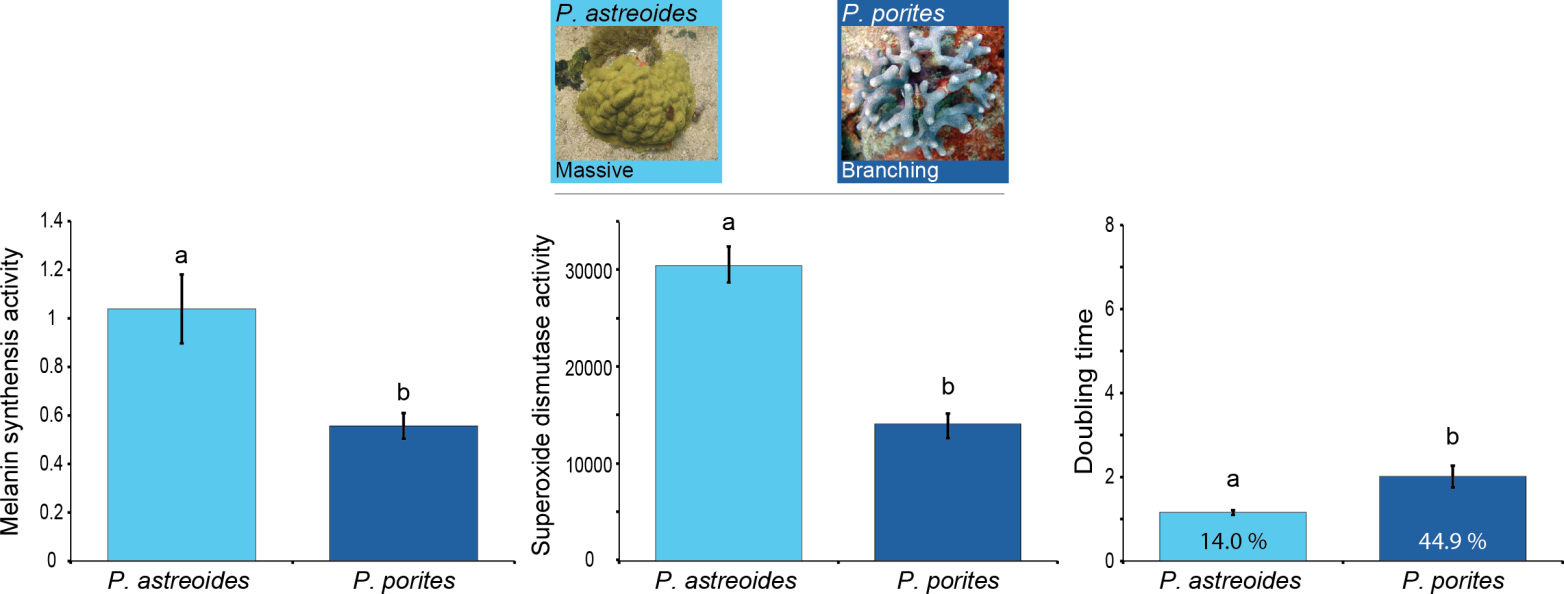
Relation between immunity and colony morphology and growth rates in corals. Mean levels of constitutive immunity among *Porites* species with different growth rates and colony morphology as determined by melanin synthesis, superoxide dismutase and the antibacterial (doubling time and percent inhibition) activity. Letters on the bars indicate significant differences (Tukey post-hoc tests at *p* < 0.05). Data presented as mean ± standard error, for melanin synthesis as Δ absorbance 490 nm mg protein^−1^ min^−1^, for superoxide dismutase as absorbance 450 nm mg protein^−1^ min^−1^, and for doubling time as hours with the percentage of inhibition inside each bar. Antimicrobial data compares growth of *Vibrio alginolyticus* when exposed to coral extract with untreated controls.

Results showed differences in baseline immunity between *Porites* spp. with different morphologies and growth rates (MANOVA Wilk’s Lambda *F*_6_ = 18.00, *p* < 0.0001; Table 1). Overall, the branching *P. porites* seems to devote more resources to growth than to immunity given its significantly lower levels of immune protein activity compared to the massive *P. astreoides* (Fig. 2). Nevertheless, there was some variation and opposing trends among the immune traits for each of these species. On one hand, *P. astreoides* seems to invest in the active elements of melanin synthesis (i.e., prophenoloxidase), rather than in the products of the pathway (melanin, 5.6 ± 0.56 mg melanin mg tissue^−1^). In contrast, *P. porites* seems to do the opposite by keeping constitutively higher melanin concentration in its tissues (20.38 ± 2.40 mg melanin mg tissue^−1^), instead of having high levels of the proteins involved in melanin synthesis. A similar trend to melanin concentration was found in bactericidal activity, where *P. porites* have higher levels of antimicrobials than *P. astreoides.*

This differential investment among immune pathways could be attributed to trade-offs between immune traits (Moret & Schimid-Hempel, 2000; Moret & Siva-Jothy, 2003; Schmid-Hempel, 2003; Adamo, Jensen & Younger, 2005; Boughton, Joop & Armitage, 2011) along with the different growth rates. The relatively less costly melanin cascade has a broader impact by contributing to various protective functions such as tissue repair, encapsulation, defense against microorganisms (Mydlarz et al., 2008; González-Santoyo & Córdoba-Aguilar, 2011) and photo-protection (Shick, Lesser & Jokiel, 1996). Antimicrobial activity, on the other hand, has a more specific function (i.e., exclusively killing pathogens) and is more costly to synthesize and use (Moret & Schmid-Hempel, 2001).

In modular organisms, like corals, growth is the result of continuous asexual production of genetically identical modules or polyps, an important characteristic that influences energy allocation (Leuzinger, Willis & Anthony, 2012) and defines life-history strategies (Darling et al., 2012). Colony growth in corals can be divided into fast-growing branching/foliose species and slow-growing crustose/massive species (Dullo, 2005). Recent observations indicate that some coral species are more resilient and more likely to overcome the impacts associated with climate change. In the Caribbean, one of the most prominent members of this group is the massive slow growing *P. astreoides* (Green, Edmunds & Carpenter, 2008; Edmunds, 2010; Palmer et al., 2011; Yakob & Mumby, 2011), which is increasing in numbers while more susceptible species, such as the branching *Acropora palmata, A. cervicornis*, and the columnar *O. annularis* and massive *O. faveolata*, are dying off (Bruckner & Hill, 2009; Miller et al., 2009; Weil, Croquer & Urreiztieta, 2009a; Weil, Croquer & Urreiztieta, 2009b). Although no study comparing changes in abundance related to disease infections between *Porites* spp. exist to our knowledge, our results suggest a mechanism (investment in constitutive immunity) for the recent survival success of *P. astreoides* from intensive bleaching events and several white plague disease outbreaks throughout the Caribbean.

### Concluding remarks

Increasing interest in understanding the levels and mechanisms of resistance of scleractinian corals is due, in part, to the increased deterioration of coral health and coral reef environments. Combinations of biological and ecological traits have been used to classify and/or determine life-history strategies in corals, and predict coral species potential for disease susceptibility (Díaz & Madin, 2010) and levels of tolerance (Darling et al., 2012; Putnam et al., 2012). Another factor that has been considered in the survival of coral species in relation to immunity is the evolutionary history. Constitutive levels of immune-related proteins have been shown to have a phylogenetic signal among Caribbean coral species with older diverged coral groups better suited to prevent infections than coral lineages that diverged more recently (Pinzón et al., 2014). However limited in species and locations, our approach takes these analyses one step further by examining how vital biological traits affect immune function among coral species. Ultimately, resistance and survivorship (fitness) of species are highly dependent on how species prioritize and distribute their resources among physiological traits. While our conclusion is restricted to two groups of Caribbean corals, the results suggest that coral immunity is an equally important variable and varies due to investment in other life-history traits such as reproductive strategy and colony growth and morphology. These findings highlight the importance of incorporating defense mechanisms (i.e., constitutive immunity levels and immune responses) to improve our ability to predict coral survival, biodiversity loss, and ecological composition of future reefs under changing conditions.

## Supplemental Information

10.7717/peerj.628/supp-1 Supplemental Information 1 Raw data with constitutive immunity levels for both *Meandrina* spp. and *Porites* spp This excel file contains the raw data used in the manuscript entitled “The link between immunity and life history traits in scleractinian corals”. The file contains two spreadsheets one for each group of species with the labels: *Meandrina* spp. and *Porites* spp. Each spreadsheet has nine columns, the first is the Sample No., then the species names and the final seven contain the values of each of the immune measures performed in this study.

## Additional Information and Declarations

### Field Study Permissions

The following information was supplied relating to field study approvals (i.e., approving body and any reference numbers): Samples were collected under the specification of research collection permits to the Department of Marine Science UPRM.
